# Understanding decision making in a food-caching predator using hidden Markov models

**DOI:** 10.1186/s40462-020-0195-z

**Published:** 2020-02-10

**Authors:** Mohammad S. Farhadinia, Théo Michelot, Paul J. Johnson, Luke T. B. Hunter, David W. Macdonald

**Affiliations:** 1 Oxford Martin School and Department of Zoology, University of Oxford, 34 Broad St, Oxford, OX1 3BD UK; 2 School of Mathematics and Statistics, University of St Andrews, The Observatory, Buchanan Gardens, St Andrews, KY169LZ UK; 3 Wildlife Conservation Research Unit, Department of Zoology, University of Oxford, Tubney House, Oxford, Oxfordshire OX13 5QL UK; 4 Wildlife Conservation Society, Bronx, NY 10460 USA; 5 School of Life Sciences, Westville Campus, University of KwaZulu-Natal, Durban, South Africa

**Keywords:** Caching behaviour, Hidden Markov models, Life-stage, Multistate animal movement, *Panthera pardus saxicolor*, Range residency, Satellite telemetry, Viterbi algorithm

## Abstract

**Background:**

Tackling behavioural questions often requires identifying points in space and time where animals make decisions and linking these to environmental variables. State-space modeling is useful for analysing movement trajectories, particularly with hidden Markov models (HMM). Yet importantly, the ontogeny of underlying (unobservable) behavioural states revealed by the HMMs has rarely been verified in the field.

**Methods:**

Using hidden Markov models of individual movement from animal location, biotelemetry, and environmental data, we explored multistate behaviour and the effect of associated intrinsic and extrinsic drivers across life stages. We also decomposed the activity budgets of different movement states at two general and caching phases. The latter - defined as the period following a kill which likely involves the caching of uneaten prey - was subsequently confirmed by field inspections. We applied this method to GPS relocation data of a caching predator, Persian leopard *Panthera pardus saxicolor* in northeastern Iran.

**Results:**

Multistate modeling provided strong evidence for an effect of life stage on the behavioural states and their associated time budget. Although environmental covariates (ambient temperature and diel period) and ecological outcomes (predation) affected behavioural states in non-resident leopards, the response in resident leopards was not clear, except that temporal patterns were consistent with a crepuscular and nocturnal movement pattern. Resident leopards adopt an energetically more costly mobile behaviour for most of their time while non-residents shift their behavioural states from high energetic expenditure states to energetically less costly encamped behaviour for most of their time, which is likely to be a risk avoidance strategy against conspecifics or humans.

**Conclusions:**

This study demonstrates that plasticity in predator behaviour depending on life stage may tackle a trade-off between successful predation and avoiding the risks associated with conspecifics, human presence and maintaining home range. Range residency in territorial predators is energetically demanding and can outweigh the predator’s response to intrinsic and extrinsic variables such as thermoregulation or foraging needs. Our approach provides an insight into spatial behavior and decision making of leopards, and other large felids in rugged landscapes through the application of the HMMs in movement ecology.

## Background

Analysing animal movement and decision making mechanisms helps understanding of inter- and intraspecific interactions, the dynamics of populations, and their distribution in space [1, 2]. In movement ecology, state-space models have been used to analyse time-indexed location data to predict the future state of a system from its previous states probabilistically via a process model [3, 4]. One particularly popular state-space model is the hidden Markov model (HMM), which can be used to describe animal movement as arising from a finite number of hidden behavioural states [5, 6]. The behavioural state process is defined as a Markov chain, i.e. the state at the next time step depends only on the current state. It is parameterized by its transition probabilities and an initial distribution [6, 7]. The observation process most often comprises the step lengths and turning angles of the animal, assumed to be driven by the underlying unobserved states [1, 7].

Decisions concerning movement across the landscape are affected by a variety of intrinsic and extrinsic factors. Age, sex and life stage, particularly range residency are key determinants of movement patterns [8, 9]. Likewise, hunger can mediate decision-making and how predators react to risk in their environments [10, 11]. In contrast, movement can vary due to extrinsic factors such as resource availability [8, 12–14], risk avoidance [15–17] and thermoregulation [18]. Various empirical studies have highlighted behavioral plasticity as a function of intrinsic and extrinsic factors in predators; however, research conceptualizing the interaction of these factors in shaping decision making across predator life stage is uncommon [8, 13].

Food caching, defined as storing and/or securing food, is an evolutionary strategy adopted by predators which can reduce kleptoparasitism or safeguard surplus food for future consumption. It is common in carnivores from red foxes *Vulpes vulpes* [19] to leopards *Panthera pardus* [20] and grizzly bears *Ursus arctos* [21]. Identifying when food is cached is useful for characterising kill sites, and consequently quantifying predator–prey dynamics [22, 23]. Although behavioural classification using the HMM trajectories are increasingly applied for different taxa, they have rarely been utilized to classify types of movement or to identify caching or kill sites [13, 24].

Here, we used HMMs on global positioning system (GPS) data collected from a food-caching predator, the Persian leopard *P. p. saxicolor* in northeastern Iran. We investigated how intrinsic and extrinsic factors interact to shape movement patterns, and how that is affected by life stage. We first examined life stage-based behavioural plasticity by estimating activity budgets during general (all relocation data) and caching (only relocation data around kills) phases of behaviour, informed using field confirmation of kill sites. We modeled a leopard’s behavioural states in relation to ambient temperature and diel patterns, two extrinsic factors associated with thermoregulation [18] and the well-established nocturnal and crepuscular activity pattern [12, 17, 25], respectively in leopards. We expected that increased hunger (days since last feeding) would trigger more searching behaviour [11], represented by long step lengths and small turning angles (i.e. fast and directed movement). Thus, we assessed the association between the behavioural responses and the likely current hunger of the individual. Finally, we estimated the activity budgets of different movement states at the two general and caching phases and investigated how they vary across the life stages. Our HMM analytical framework helps us to unfold the association between energetic demands and behavioural decision-making which has implications for predator-prey dynamics, resource selection and even human wildlife interaction.

## Methods

### Study species and area

We studied leopards in Tandoureh National Park in north-eastern Iran (ca. 20 km from the Turkmenistan border). The park has been protected since 1968 and covers 355 km^2^. It ranges in elevation from from 1000 to 2600 m and is characterized by mountains with scattered juniper trees *Juniperus* sp. Prey availability in Tandoureh is affected by the national park boundaries, with wild medium-sized prey available only inside the park, whereas domestic animals are found exclusively outside the park where human settlements are located. The only exception is wild pig *Sus scrofa*, which is occasionally found in rural areas, outside the national park [26].The diet of leopards in the study area is composed of around 80% of wild ungulates and 20% domestic animals [23]. Using the auto-correlated Kernel density estimation method, we estimated a mean home range of 103.4 ± SE 51.8 km^2^ for resident male leopards [27]. If three-dimensional topographic estimation to account for vertical relief is considered, leopards’ home range will increase up to 38%, making them larger than has hitherto been observed in other studies of Asian leopards [28].

### Leopard capturing and handling

We captured leopards with Aldrich foot-snares modified extensively to reduce risk of injury and remotely monitored with VHF trap transmitters (Wildlife Materials, Inc., Illinois, USA) every 1–2 h. A wild pig carcass was used as bait, normally hanging from a tree or rock. Traps were also deployed along trails leading to the baits. In summer, we deployed traps along trails leading to water sources, sometimes without bait (see [29] for more details).

We immobilized leopards using a combination of ketamine 10% (Alfasan, Nederland BV) 2 mg/kg, medetomidine HCl 20 mg/ml (Kyron Laboratories (Pety) Ltd., Johannesburg, South Africa) 30 μg/kg and butorphanol 0.2 mg/kg (Torbugesic®, Fort Dodge Animal Health Fort Dodge Animal Health, Iowa 50,501 USA) delivered intramuscularly with a dart gun (Daninject, Denmark) using a 1.5 ml dart.

We used Iridium GPS collars (LOTEK Engineering Ltd., Newmarket, ON, Canada). Each collar incorporated a drop-off buckle with a timer set to 52 weeks since deployment. Each animal’s age was estimated based on dental features. Anesthesia lasted for 44 to 60 min, followed by reversal using atipamazole (3 times the medetomidine dosage) and nantroxan (the doses equal to butorphanol), injected intramuscularly (see [29] for more details).

### Cluster investigation

**Fig. 1:**
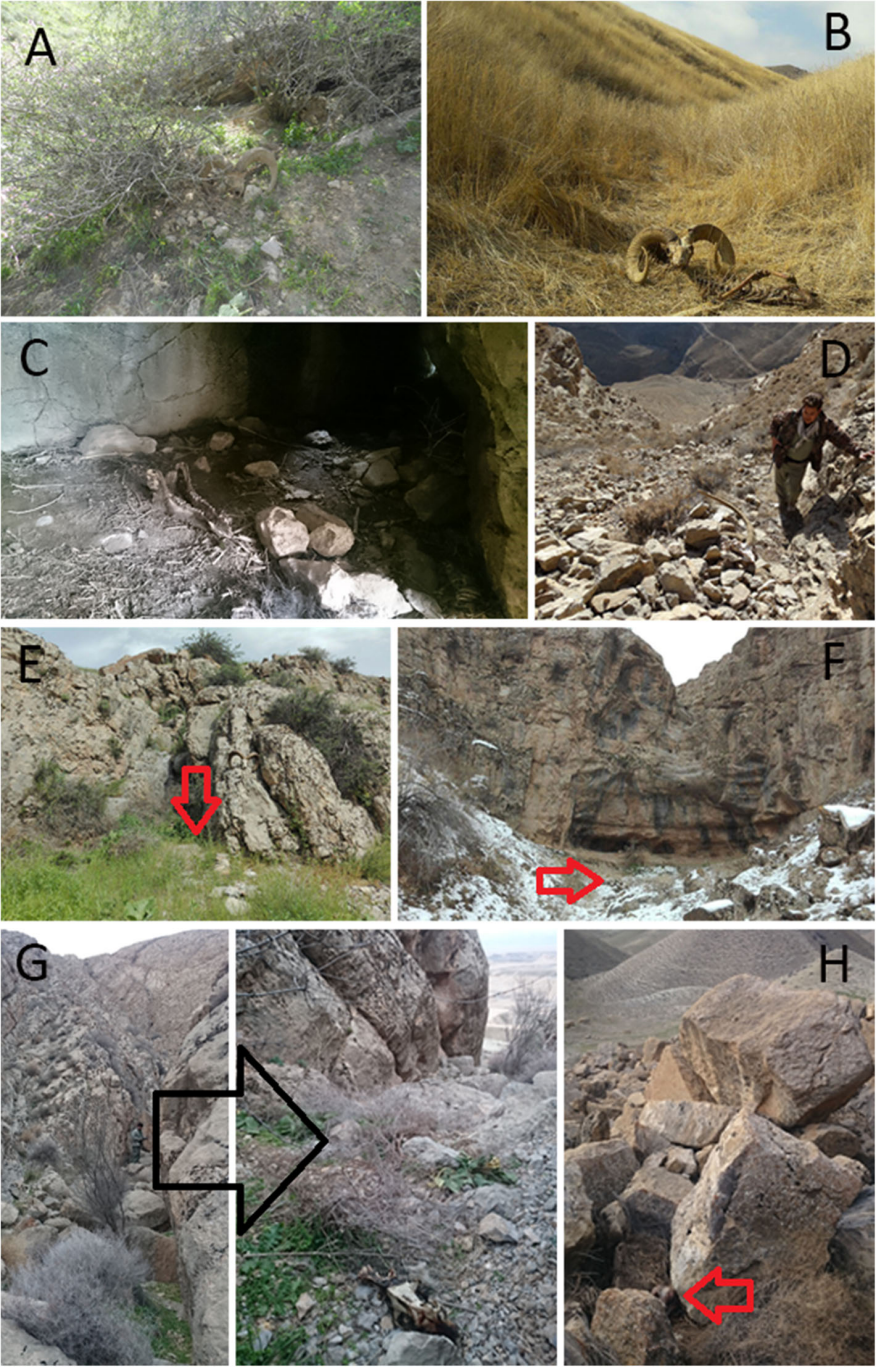
Different types of kill hoarding, known as caching behaviour, in Persian leopards in Tandoureh National Park and surrounding areas along the Iran-Turkmenistan borderland. **a** an urial ram under a tree, **b** an urial ram in dense vegetation, **c** a wild pig inside a rocky hollow, **d** a bezoar goat among cliffs in high elevations, **e** & **f** urial rams under cliffs at the end of valleys, **g** left image shows the position of a dog, which is zoomed in the right image, next to communal lands in the far background, and **h** a dog concealed among cliffs (Photos by M. Farhadinia, K. Hobeali, P. Behnoud, P. Moghadas and S. Firouzi)

In Tandoureh, leopards exhibit short-term food caching, frequently among rocks or dense vegetation (Fig. 1) which yield spatially aggregated GPS fixes, or clusters. Accordingly, GPS clusters - defined as locations where leopards remained overnight (between 18:00 and 06:00 h) within a radius of 200 m - were investigated for possible kill remains (see [23] for more details).

We followed Knopff et al. [22] who recommended recording fixes every 3 h to enable the identification of clusters. To increase success rates of locating clusters and safeguarding a balance between battery life and acquisition rate [30] fixes were taken hourly during the last week of each month. Also, a ‘virtual fence’ option enabled us to upload the protected area’s boundary, so that when leopards left the defined area, fix rate could be increased to hourly. It enabled us reliably to detect kills outside the national park where prey generally have a smaller body mass (i.e., domestic animals) and scavengers, such as herding and stray dogs *(Canis familiaris)*, golden jackals *(Canis aureus)*, and striped hyenas *(Hyaena hyaena)*, are more abundant.

We discarded fixes from the first 4 days post-collaring, associated with the earliest known kill made by the leopards after collaring, to avoid the bias due to possible abnormal movements immediately after collaring [31]. We identified potential clusters visually using Google Earth 5 (Google Corporation 2009) and a web-based map system for displaying telemetry data (webservice.lotek.com) in a 6–8-day timeframe. Potential GPS cluster locations were then uploaded on a hand-held GPS device (Garmin GPS62S, Garmin International, Olathe, Kansas) to enable ground crews of ≥2 people to systematically search within a radius of at least 100 m from each cluster location for at least 20 min, following Knopff et al. [22]. Prey remains were thoroughly investigated to identify prey species. Potential cluster locations were visited between September 2014 and May 2017, when the last leopard’s collar stopped transmitting relocation data via the Iridium satellite. Twelve locations were discarded as not being accessible because of extreme weather at high elevations, leaving a total of 310 locations that were investigated as potential clusters. If prey remains were found, the cluster was categorized as a “caching site”, otherwise as a “bed site”.

### Extrinsic and intrinsic covariates

To investigate the drivers of movement behaviours, we derived two sets of extrinsic and intrinsic covariates for each GPS fix, including ambient temperature, diel period and hunger level. The collars recorded time of day and temperature for each fix, the latter was used as a proxy for the ambient air temperature due to the strong correlation between collar and ambient temperatures [32].

Hunger level was defined as days since last feeding [10]. We acknowledge that we may have missed small prey (< 8 kg), because cluster duration, dependent on prey size, can affect success of locating kills [33, 34]. Nonetheless, we attempted to account for the biases that ignoring small prey items can potentially introduce to the quantified hunger level by two means. First, ungulates’ newborn lambs, partially comprise the spring diet of Persian leopards in Tandoureh [23], are quickly consumable prey. We therefore excluded the relocation data during the first 2 months following the lambing season (1 May through 30 June).

Second, leopards are known to use small prey [35, 36]. These are difficult to detect in cluster investigations due to their rapid consumption. We therefore quantified the role of small-bodied prey (rodents, lagomorphs, and birds) using fecal analysis of leopard scats (see Additional file 1 for more details). We estimated the frequency of occurrence, defined as the percentage of total scats in which a food item was found, as 90.4% for medium-sized mammals (Additional file 1: Table S1). Therefore, the time since last feeding of a medium-sized prey can reliably represent hunger level as small-bodied animals were likely to make a minor contribution to the diet of leopards in Tandoureh.

We also investigated how the effect of these intrinsic and extrinsic covariates on behavioural decisions depends on an individual’s life stage. Accordingly, each individual’s life stage was assigned based on the criterion of range residency behavior, as evidenced by plots of the semi-variance in positions as a function of the time lag separating observations (i.e., variograms) with a clear asymptote at large lags [37, 38], resulting in assigning resident or non-resident to collared individuals. The visual verification of range-residency via variogram analysis was conducted using the R package ‘ctmm’ version 0.4.0, following the workflow described by Calabrese et al. [37].

### Multistate movement analysis

We used hidden Markov models (HMMs) to infer behavioural states, and the corresponding movement parameters, from the movement data. The crucial requirements for movement data to be suitable for HMMs are that measurement error in positions should be negligible and that there should be a regular sampling unit [1]. In our previous paper, we detected neither erroneous fixes nor spikes in movement [27], using a script developed by Bjørneraas et al. [31] implemented in the R environment for statistical computing [39]. The package “moveHMM” [7] in R was used for fitting the HMMs to the movement tracks.

The collars were set up to alternate between a frequency of one fix per hour and one fix every 3 h, as described in the ‘Leopard capturing and handling’ section. To accommodate the assumption of HMMs that the sampling rate is regular, we therefore split the data set into two: one for 1-h fixes (16,118 locations), and one for 3-h fixes (7875 locations). The HMM parameters are dependent on the sampling scale, so for each sampling scale, two sets of models were separately fitted for the two individual life stages, i.e. residents and non-residents.

HMMs require that the number of behavioural states be chosen before fitting the model. In principle, it is possible to fit several models with different numbers of states, and compare them using e.g. the Akaike Information Criterion (AIC) to identify the better formulation. However, simple statistical criteria have been shown to select very large numbers of states, to the detriment of biological interpretability [40, 41]. We considered HMMs with three behavioural states, based on our prior knowledge of another caching felid, puma *Puma concolor*, suggesting that 3-state models are generally statistically well-supported and biologically interpretable. They are resting, moderately active and traveling modes, differing in step lengths and turning angles [13].

We modeled the step lengths with gamma distributions, and the turning angles with von Mises distributions. With the HMM, we estimated several behavioural states, characterized by different distributions of step lengths and turning angles. We used the Viterbi algorithm to predict the most likely sequence of behavioural states under the fitted model, i.e. to assign a state to each observed step [6]. From the estimate state sequence, we derived the activity budgets of the animals, i.e. the proportion of time spent in each behavioural state. We compared the estimated activity budgets for all relocation data to those obtained for the caching periods only (relocation data around kill sites). Kill sites were identified during field investigations (see ‘Cluster investigation’ for more details) and included all locations within a radius of 200 m of kill remains, because of the predators’ tendency to leave cache sites to visit rest sites which may be some distance away and then return to the site to feed [42, 43].

We used covariates in the transition probabilities of the state process, to investigate the effect of environmental and individual-specific variables on behavioural decisions. We applied a stepwise procedure to select the covariates, and fitted four models to each data set based on life stage: (1) no covariates, (2) time of day, (3) time of day + temperature, and (4) time of day + temperature + hunger. We used the AIC to select the best formulation, from the candidate models. To ensure that the effect of the time of day is cyclical over 24-h periods, we used trigonometric link functions, as described by Leos-Barajas et al. [44]. We included the effect of the three covariates described above on the transition probabilities of the state process, as described in Michelot et al. [7]. We finally investigated the activity budgets of the leopards, i.e. the estimated proportion of time spent in each state, during general and caching phases based on leopard life stage.

We examined the goodness-of-fit using the pseudo-residuals of the fitted model. The pseudo-residuals should be approximately normally distributed if the estimated densities of step lengths and turning angles fit the data well [5]. We considered only the pseudo-residuals for the step lengths, because the definition of turning angle pseudo-residuals is arbitrary, due to the circularity of the variable [41].

## Results

Between September 2014 and May 2017, six leopards (5 males, 1 female) were collared and monitored in Tandoureh (Table 1). GPS collars collected data for between 54 and 368 days per individual, representing a total number of 56.7 monthly leopard study periods. Our GPS locations represented 1702 leopard-days (283.7 ± SD 124.4 days/leopard; Table 1), with a high overall fix success rate (mean 85.0% ± SD 7.6). Importantly, 17.9 ± 7.3% of collaring days overall (varying between 0.0 and 43.8% for different individuals) were located outside the park, five of the six collared leopards had some degree of home range overlap with communal lands including villages, farmland and pastures (Fig. 1).

**Table 1:** Details of Persian leopards collared in Tandoureh National Park, northeastern Iran (2014–2017). M1 (old male) showed a mixed ranging pattern. He showed resident behavior until almost 5.5 months after collaring when his semi-variance increased and he started his excursions outside the park along the borderland’s communities with regular returns to the national park

Leopard Name/ID	Sex/age	Capture date	Last fix	Number of days	Life stage
M1/Borzou	M/+ 10	5.2.2015	4.2.2016	368	Resident/non-resident
M2/Bardia	M/8–10	3.10.2014	30.9.2015	362	Resident
M3/Borna	M/5–6	28.9.2014	27.9.2015	364	Resident
M4/Tandoureh	M/7–10	16.8.2016	1.04.2017	228	Resident
F5/Iran	F/2–3	6.12.2015	29.1.2016	54	Non-resident
M6/Kaveh	M/3–4	4.9.2015	26.8.2016	326	Non-resident

Three males (M2/Bardia, M3/Borna and M4/Tandoureh) exhibited constrained space use as resident individuals, based on a clear asymptote in variograms (Table 1 and Additional file 1: Figure S1). In contrast, the two youngest leopards (F5/Iran and M6/Kaveh) lacked asymptotes, showing a non-residency pattern. M1/Borzou (an old male) showed a mixed ranging pattern. Following 5.5 months of residency behavior inside the national park, he started his excursions outside the park along the borderland’s communities with regular returns to the national park which was associated with an increase in his semi-variance (Additional file 1: Figure S1). We therefore split the relocation data from M1/Borzou to two stages of residency (5.5 months) and non-residency (6.5 months). We present results for two sampling frequencies, i.e. 1 and 3-h.

### 1-h data sampling interval

We fitted four 3-state models for each life stage, with different covariate dependencies on transition probabilities. The models with all covariates (time of day + temperature + hunger) were favored by the AIC (Table 2) for both life stages, and it is the model we describe in the rest of the analysis. The model output is presented in Table 2.

**Table 2:** Number of estimated parameters (K), AIC, and ΔAIC (compared with best model) for 3-state HMMs with different covariate dependences during general phase for residents and non-residents, based on 1-h interval dataset. The effect of the time of day is cyclical over 24 h. For both data sets, the model with all three covariates was selected by the AIC

	K	AIC	ΔAIC	Model weight
Resident individuals				
Time of day + Temperature + Hunger	47	6257.7	0	1
Time of day + Temperature	41	6285.2	27.5	0
Time of day	35	6312.5	54.8	0
No covariate	23	6370.1	112.3	0
Non-resident individuals				
Time of day + Temperature + Hunger	44	5849.7	0	1
Time of day + Temperature	38	5900.7	50.9	0
Time of day	32	5982.3	81.6	0
No covariate	20	6176.0	193.7	0

**Fig. 2:**
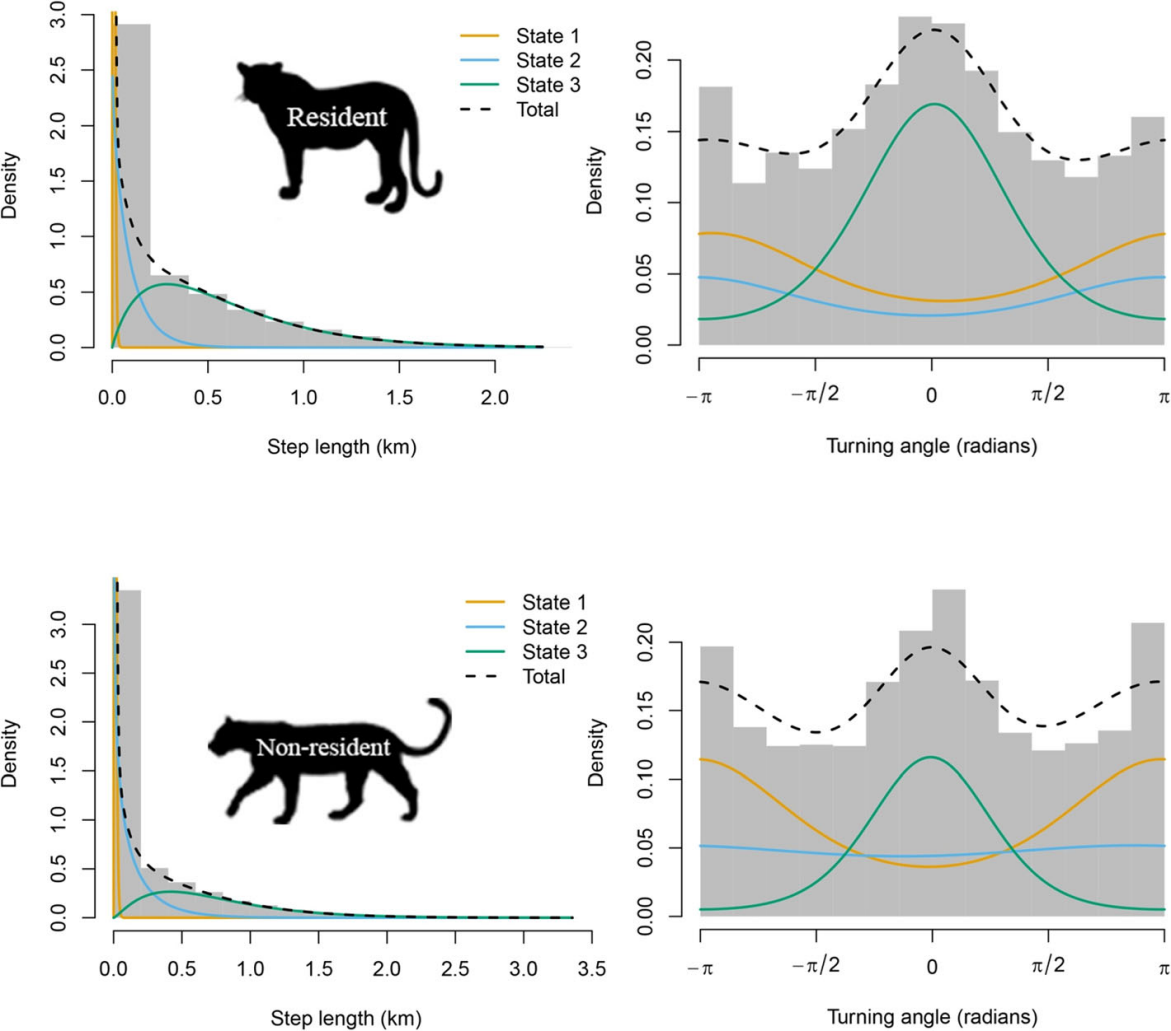
Histograms of observed step lengths (left) and turning angles (right) in the 1-h data set for general phase of resident and non-resident leopards. The colored lines are the estimated densities in each state, and the dotted black line is their sum

The states were associated with three distinct types of movement patterns (the states can be said to be distinct in that the confidence intervals for their attributes did not overlap, Table 3). State 1 captured short step lengths and very variable turning angles, which corresponds to slow and undirected movement (resting mode). State 3 captured long step lengths and small turning angles (concentrated at zero), i.e. fast and directed movement (traveling mode). State 2 was intermediate between states 1 and 3, and captured moderately fast and directed movement (moderately active mode; Fig. 2).

**Table 3:** Estimates and 95% confidence intervals of the movement parameters, for the selected 3-state models. The step lengths are modeled with a gamma distribution, and the turning angles with a von Mises distribution based on 1-h data set for resident and non-resident collared leopards

	State 1 (resting)	State 2 (moderately active)	State 3 (traveling)
Resident			
Step mean (km)	0.006 (0.006,0.007)	0.095 (0.080,0.113)	0.600 (0.570,0.625)
Step SD (km)	0.005 (0.004,0.005)	0.100 (0.080,0.121)	0.430 (0.410,0.451)
Angle mean (radians)	−2.97 (−2.80,-3.15)	3.10 (2.82,3.84)	0.04 (−0.03,0.10)
Angle concentration	0.46 (0.38,0.55)	0.41 (0.27,0.57)	1.11 (1.00,1.22)
Non-resident			
Step mean (km)	0.009 (0.008,0.009)	0.147 (0.126,0.172)	0.740 (0.705,0.777)
Step SD (km)	0.007 (0.007,0.008)	0.180 (0.154,0.210)	0.489 (0.467,0.513)
Angle mean (radians)	3.10 (3.00,3.20)	2.74 (1.31,3.79)	−0.02 (−0.07,0.03)
Angle concentration	0.57 (051,0.64)	0.08 (0.02,0.17)	1.56 (1.40,1.73)

The HMM framework provided strong evidence for clear difference in the estimates of movement parameters between the two life stages. Thus, regardless of the behavioural states, mean step length was shorter in resident comparing to non-resident leopards (Table 3). For example, in state 3 (traveling mode), the resident leopards moved an average of 600 m per hour (95% CI 570–625 m), whereas non-residents moved an average of 740 m per hour (95% CI 705–777 m). The estimates of all movement parameters, as well as the corresponding 95% confidence intervals, are given in Table 3. The difference between life stages is less evident for turning angles (Table 3).

**Fig. 3:**
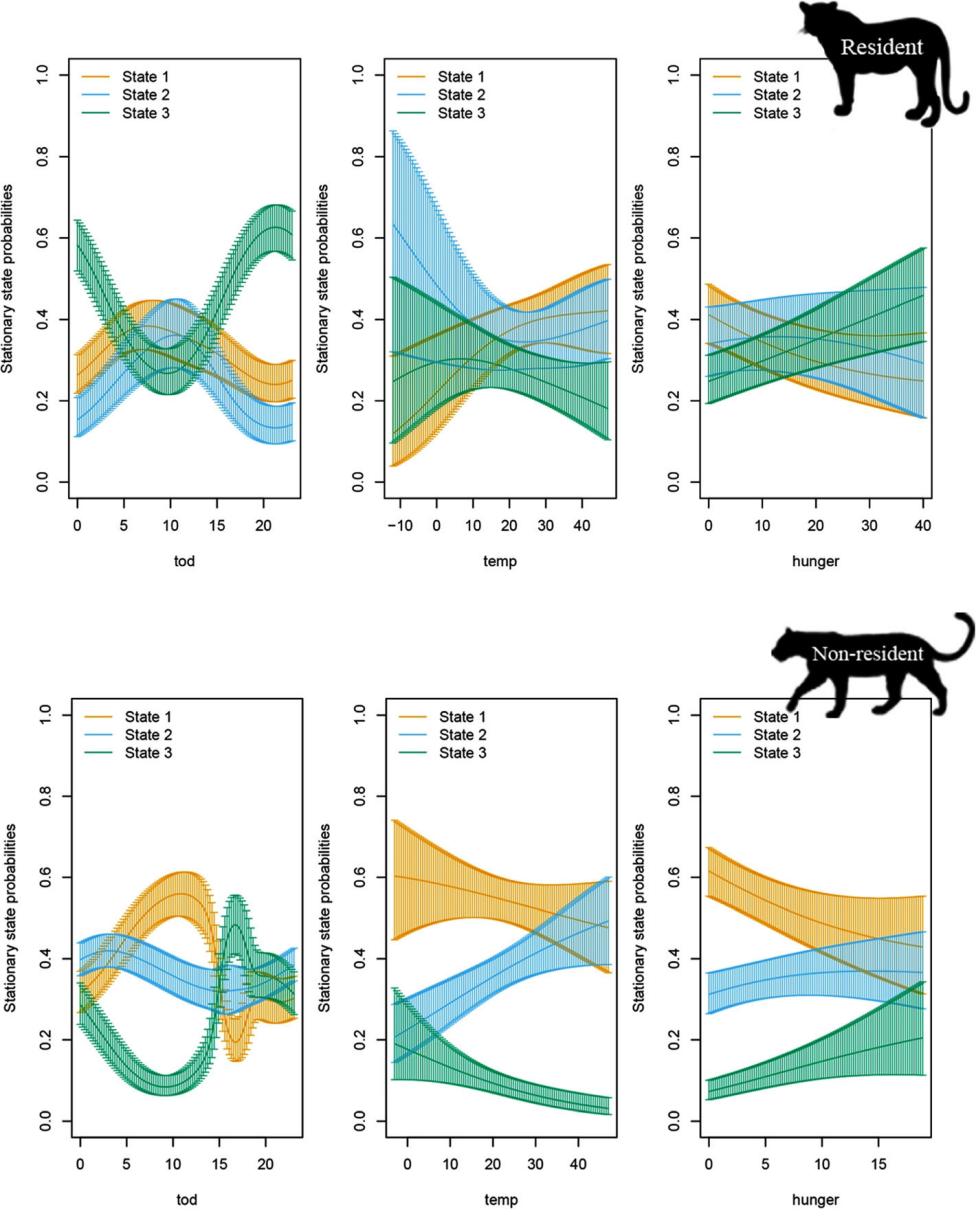
Stationary state probabilities for the 1-h data set during general phase for of a) resident and b) non-resident individuals, as functions of the three covariates: time of day (left), temperature (middle), and hunger (right). The vertical lines give point wise 95% confidence intervals. States are: 1 = slow and undirected movement, 2 = moderately fast and directed movement and 3 = fast and directed movement

There were also clear differences in how the behavioural states were related to covariates across the two life stages. The effects of temperature and hunger were much less clear for residents compared with non-residents. Only time of day had a strong effect on the state probabilities (Fig. 3): the leopards are more likely to be in state 3 (fast directed movement in traveling mode) in the evening and states 1 and 2 in the morning, suggesting that resident leopards use the darker hours for patrolling. In contrast, non-resident leopards were more likely to show directional states of movement (2 and 3) at high hunger levels, suggestive of searching for prey. Similar to resident leopards, they are also more likely to patrol in darker hours (Fig. 3).

The quantile-quantile plot of the step length pseudo-residuals suggests some lack of fit in the tails (Additional file 1: Figure S2). However, there was little residual autocorrelation, so the 3-state model appeared to successfully capture the autocorrelation in the observed step lengths.

### 3-h sampling interval

We repeated the analysis for the data set with 3-h time intervals, split between the two life stages. As for the other data set, the model with all covariates was selected by the AIC (Additional file 1: Table S2), illustrating the three movement states with similar step length and turning angles as the 1-h time intervals (Additional file 1: Figure S3). Note that, although they describe similar types of movement patterns, the states of the model fitted to the 1-h data set cannot be easily compared with those of the model fitted to the 3-h data set. Indeed, discrete-time movement metrics (such as step lengths and turning angles) depend strongly on the time step, and cannot be compared across temporal scales [45]. The estimates and 95% confidence intervals of the movement parameters are given in Additional file 1: Table S3, showing that the step lengths and turning angles are different at different time scales of sampling (Table 3 and Additional file 1: Table S3).

The stationary state probabilities are displayed in Additional file 1: Figure S4 as functions of the covariates. The effect of the time of day is similar to what was observed in the 1-h data set for both life stages: state 3 was more likely in the evening whereas states 1 and 2 more likely in the morning. Nonetheless, the wide confidence bounds for all stationary state probabilities as functions of hunger and time of day suggested that the leopard behavioural states do not show distinctive response to these two covariates at 3-h sampling intervals for both life stages (Additional file 1: Figure S4). As in the 1-h model, there was some lack of fit for extreme step lengths, and very little residual autocorrelation (Additional file 1: Figure S5).

### Caching behavioural patterns

Out of 310 investigated potential clusters, prey remains were found in 130 (41.9%). We found 10 prey items assigned to three categories: wild ungulates (urial, bezoar goat and wild pig), domestic animals (dog and sheep) and small animals, such as Indian crested porcupine, red fox, raptors, pigeon *(Columba livia)* and chukar partridge *(Alectoris chukar)*. We excluded those small kills because they are less likely to create a food-caching behaviour. The leopards spent an average of 51.9 ± SD 32.3 h at each caching site (within a radius of 200 m of kill remains) of medium-sized prey. At 30.1% of the sites, leopards undertook brief (3.0 ± 1.0 h) excursions away from the feeding area to which they intermittently returned.

There was strong evidence that the temporal budget of behavioural states for 1-h sampling interval depended on life stage. During the general phase, residents spent more time in state 3 (fast, directed movement in traveling mode, the green curves were higher) whereas the non-residents spent more time in state 1 (slow undirected movement in resting mode, the orange curves were higher; Table 4 and Fig. 3). Caching sites predominantly corresponded to state 1 (slow and undirected movement) for both residents and nonresidents (Table 4).

**Table 4:** Temporal budget of HMM behavioural states of the Persian leopards based on GPS relocation data at two phases based on 1-h data sampling interval for resident and non-resident collared leopards. The behavioural budget is analysed during general and caching phases. States are: 1 = slow and undirected movement, 2 = moderately fast and directed movement and 3 = fast and directed movement

Behavioural state	Caching phase			General phase		
	State 1 (resting)	State 2 (moderately active)	State 3 (traveling)	State 1 (resting)	State 2 (moderately active)	State 3 (traveling)
Resident individuals	58%	31%	11%	33%	20%	47%
Non-resident individuals	68%	24%	7%	44%	30%	26%

**Fig. 4:**
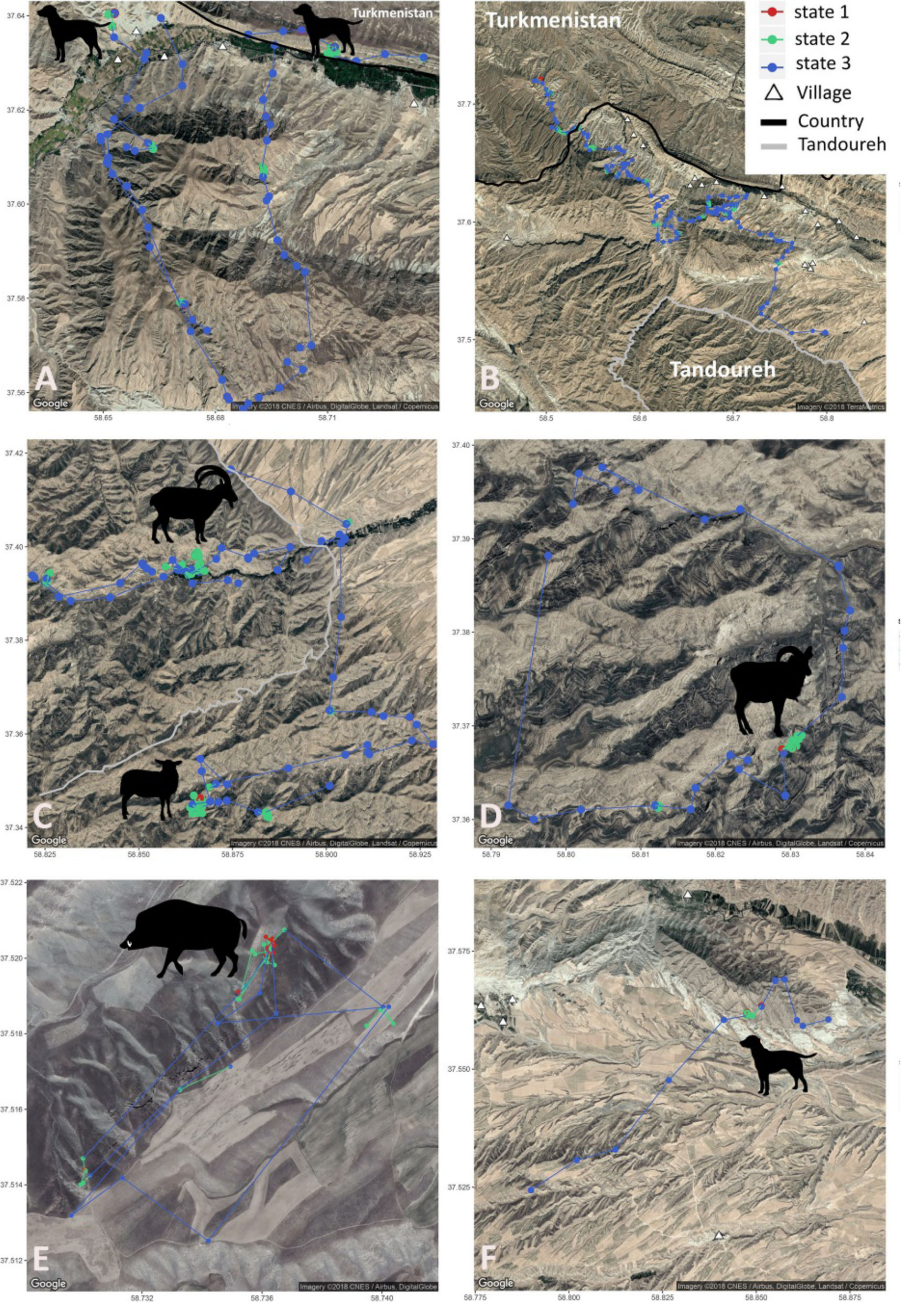
Example tracks of the Persian leopards at 1-h sampling intervals, colored by the most likely behavioural states obtained with the Viterbi algorithm. **a** M1 (old male/non-resident) killing two dogs on communal lands outside the national park 25/12/2015 to 07/01/2016; **b** dispersal stage of M6 (young male//non-resident) traveling from the national park through communal lands to Turkmenistan 15/02/2016 to 02/03/2016; **c** two caching sites for M3 (resident male), with a bezoar goat and a domestic sheep, the latter outside the national park; **d** a 5 days ranging of M2 (resident male), including killing an adult urial ram 26/12/2015 to 1/3/2015; **e** A zoomed in part of the caching behaviour of M6 (non-resident male), at a wild pig kill on communal lands adjacent to the national park; and **f** hunting a dog by M6 (non-resident male), amidst villages

At longer sampling intervals (3-h), there was no marked difference in time budget of behavioral states at each level of caching or general phases. Accordingly, state 1 dominated the caching phase while it was the least common in the general phase (Additional file 1: Table S4). Figure 4 demonstrates state decoding, with examples of tracks of leopards in different stages (dispersal, home range patrolling, and raiding stock animals) as well as illustrating the fine scale movement trajectory at a caching site (Fig. 4e).

## Discussion

The HMM approach used in this study allowed us to elucidate the processes regulating the movement of a food-caching predator, the Persian leopard. We examine movement patterns at two nested scales, i.e. the ‘general’ and ‘caching phases’. We find strong evidence for a life stage effect on the stationary state probabilities and their associated time budget at both scales.

Our findings provided new insights into the effect of life stages and their different movement patterns [8, 12, 46]. Resident leopards were more often mobile (state 3; traveling mode with fast directed movement) whereas non-resident leopards spent most of their time in less mobile mode (state 1; resting mode with slow and undirected movement). Residents need to maintain their home range and defend access to mates regardless of foraging or thermo-regulatory requirements, forcing them to adopt an energetically more costly mobile behaviour. In contrast, non-residents need to ensure that their foraging needs are met while avoiding conspecifics. Thus, while meeting their foraging needs in high risk lands through engaging in depredation on domestic animals or scavenging [23], adopting a less mobile strategy, both during caching as well as general phases, enables non-residents to minimize the risk.

Although environmental covariates (ambient temperature and diel period) and ecological outcomes (predation) strongly affected behavioural states in non-resident leopards, the response in resident leopards was not clear, except for temporal patterns, which was consistent with the nocturnal and crepuscular behaviour of leopards [17, 25]. Resident leopards (only males in this study) were more active in the hours of darkness, likely to use the night time to patrol their home range without being spotted by conspecifics or prey (Fig. 3). There was no evidence for an effect of either hunger or ambient temperature on their activity, suggesting that movement is less predictable by their need for foraging or thermo-regulation.

Non-resident leopards, in contrast, tended to remain inactive during the daytime and warm temperatures (state 1/resting mode) whereas they shifted to more mobile states in colder and darker times. These diel activity and temporal shifting behavioural patterns could be explained by thermo-regulatory strategies [18] as well as by leopards’ making behavioural adjustments to minimize the risk of detection by competitors, i.e. conspecifics and humans, and their prey [17, 25]. In contrary to residents’ response to hunger, non-resident leopards tended to travel more slowly with undirected, convoluted orientation after a meal while they shift to travelling mode (state 3) when hunger increases.

Similarly, the temporal budget of these leopards was affected by life stage. In Tandoureh, leopards’ caching strategy is a combination of hoarding the kill within available microhabitat features (Fig. 1) and with strictly constrained movement patterns around the kill, i.e. small step lengths and high turning angles usually with occasional short bouts of leaving and returning to caches (Fig. 4e). This encamped state (resting mode) was more common in non-resident comparing to resident leopards when caching.

Importantly, in the absence of other large carnivores, kleptoparasitism by conspecifics can still be a major source of losses [20]. The high population density (5.6 individuals/100 km^2^ [47]) and large home range overlap between neighboring conspecifics in Tandoureh [27] make caching behaviour important for avoiding this [20, 48].

The HMM approach somewhat underestimated the density of very short and long step lengths. The lack of fit of the step length distributions would be of concern for the simulation of realistic movement trajectories, and it could be remedied with the inclusion of additional states in the model. However, in this study, we focused on the classification of the tracks into movement states, so we favored a model with fewer states and a clear biological interpretation [41] based on prior knowledge of caching felids [13]. Also, our sample size of individual leopards was small and male-biased. Therefore, future HMM studies based on larger sample sizes may be able to refine diagnostic tools of this kind to accommodate inter-sexual differences, individual variability and the influence of life history on energetics. Finally, given the clear shift in behaviour between general and caching phases, the HMMs using readily transmitted GPS relocation data can objectively identify and visualize kill clusters to direct field efforts, particularly in remote landscapes.

## Conclusions

Our study demonstrates that plasticity in predator behaviour depending on life stage may reflect a trade-off between acquiring energetic rewards and avoiding risks associated with conspecifics, human presence and maintaining home range. Range residency in territorial predators is energetically demanding and can outweigh the predator’s response to intrinsic and extrinsic variables such as thermoregulation or foraging needs. Our approach provides an insight into the spatial behavior and decision making of leopards, and other large felids in rugged landscapes through the application of the HMMs in movement ecology.

## Supplementary information

**Additional file 1.** Understanding decision making in a food-caching predator using hidden Markov models.

## Data Availability

All locational data are publicly available on Movebank: https://www.movebank.org/panel_embedded_movebank_webapp. Project: Persian leopard Tandoureh Iran (accession number 270329098). All the data are available for download as .csv file.

## Abbreviations

AIC: Akaike Information Criterion
GPS: Global positioning system
HMM: Hidden Markovian model
