# Supplementary material for: Understanding decision making in a food-caching predator using hidden Markov models
Source: Mov Ecol. 2020 Feb 10;8:9. doi: 10.1186/s40462-020-0195-z (PMC7011357; doi:10.1186/s40462-020-0195-z)
Supplement: Supplementary file 1 — Additional file 1. Understanding decision making in a food-caching predator using hidden Markov models. [file 40462_2020_195_MOESM1_ESM.docx]

**Additional file 1**

**Understanding decision making in a food-caching predator using hidden Markov models**

Mohammad S. Farhadinia^1^ *, Théo Michelot^2^, Paul J. Johnson^6^, Luke T.B. Hunter^3,4^ and David W. Macdonald^6^

^1^ Oxford Martin School and Department of Zoology, University of Oxford, 34 Broad St, Oxford OX1 3BD, Oxford, UK

^2^ School of Mathematics and Statistics, University of St Andrews, The Observatory, Buchanan Gardens, St Andrews KY169LZ, UK

^3^ Wildlife Conservation Society, Bronx, NY, 10460, USA

^4^School of Life Sciences, Westville Campus, University of KwaZulu-Natal, Durban, South Africa

^6^ Wildlife Conservation Research Unit, Department of Zoology, University of Oxford, Tubney House, Oxfordshire, OX13 5QL, Oxford, UK

* Corresponding author email: [mohammad.farhadinia@zoo.ox.ac.uk](mailto:mohammad.farhadinia@zoo.ox.ac.uk)

## Dietary analysis based on fecal sampling

During the monitoring of collared leopards, we collected leopard scats in Tandoureh National Park. To minimize the chance of misidentification with those of sympatric large carnivores’ such as wolf or hyena, only unambiguous scats with characteristic shape (cat-like segmentation and pointed ends) and ≥25 mm in diameter defecated at leopard scrapes were collected.

All scats were individually stored in plastic bags and later analyzed using techniques standardized by Mukherjee et al. [1]. In brief, samples were washed in running water through a fine-mesh sieve to remove surface oil and to separate the hair from other undigested organic matter. All remains were air-dried and 20 random hairs per sample were compared microscopically with a reference hair collection [2]. Rodents were also detected based on their bones and skulls, while feather and scale were useful to record birds and reptiles, respectively. We assumed that presence of leopard hair in the scat which with respect to lack of claws or bones support that the fecal sample belongs to leopard, digesting hair during self-grooming.

For statistical quantification, we determined frequency of occurrence, defined as the percentage of total scats in which a food item was found. Using the frequency of occurrence may exaggerate the importance of smaller-sized prey in a predator’s diet, since the consumption of small prey generally produces more indigestible matter in scats, but represents less biomass consumed [3,4].

Out of 141 scats collected, 63 samples (44.7%) contained leopard hair, indicating that the associated scat samples belong to leopards which have digested hair during self-grooming. We used only those scats containing leopard hair for the dietary analysis. The leopard scats contained 9 different species (assuming that all bird and rodent remains belonged to single species), and diet was dominated by medium-sized ungulates (Table S1). Excluding non-food items and non-nutritive plant materials, 90.4% total food items were medium-sized mammals.

Table S1 Frequency of prey items of the leopard based on fecal analysis in Tandoureh National Park, northeastern Iran (2014-2017)

| **Food item** | N | Mean body weight (kg) | Frequency of Occurrence (%) |
| --- | --- | --- | --- |
| *Medium-sized food items* |  |  |  |
| Urial | 38 | 46.5 | 60 |
| Bezoar goat | 19 | 42.1 | 30 |
| Wild pig | 1 | 71.5 | 2 |
| Livestock | 6 | 50 | 10 |
| Domestic dog | 2 | 35 | 3 |
| *Small-sized food items* |  |  |  |
| Common fox | 1 | 3 | 2 |
| Afghan pika | 3 | <0.5 | 5 |
| Porcupine | 0 | 11 | 0 |
| Rodents | 2 | <0.5 | 3 |
| Birds | 1 | <0.5 | 2 |
|  |  |  |  |
| **No. of food items** | **73** |  |  |
| **No. of scats** | **63** |  |  |
| **No. of food items/scat** | **1.16** |  |  |

Table S2 Number of estimated parameters (K), AIC, and ΔAIC (compared with best model) for 3-state HMMs with different covariate dependences during general phase for residents and non-residents, based on 3-hour interval dataset. The effect of the time of day is cyclical over 24 hours. For both data sets, the model with all three covariates was selected by the AIC.

|  | K | AIC | ΔAIC | Model weight |
| --- | --- | --- | --- | --- |
| *Resident individuals* | | | |  |
| Time of day + Temperature + Hunger | 47 | 12138.96 | 0 | 1 |
| Time of day + Temperature | 41 | 12193.36 | 54.4 | 0 |
| Time of day | 35 | 12228.24 | 89.28 | 0 |
| No covariate | 23 | 12385.19 | 246.23 | 0 |
| *Non-resident individuals* | | | |  |
| Time of day + Temperature + Hunger | 44 | 3539.9 | 0 | 1 |
| Time of day + Temperature | 38 | 3611.8 | 71.9 | 0 |
| Time of day | 32 | 3624.6 | 84.6 | 0 |
| No covariate | 20 | 3689.1 | 149.1 | 0 |

Table S3 Estimates and 95% confidence intervals of the movement parameters, for the selected 3-state models. The step lengths are modeled with a gamma distribution, and the turning angles with a von Mises distribution based on 3-hour data set for resident and non-resident collared leopards.

| *Resident* | | | |
| --- | --- | --- | --- |
|  | State 1 (resting) | State 2 (moderately active) | State 3 (traveling) |
| Step mean (km)  Step SD (km)  Angle mean (radians)  Angle concentration | 0.007 (0.007,0.008)  0.006 (0.005,0.006)  -3.14 (-3.23,-3.05)  0.51 (0.46,0.57) | 0.099 (0.086,0.114)  0.109 (0.092,0.129)  3.06 (2.83,3.27)  0.27 (0.18,0.37) | 0.629 (0.605,0.655)  0.450 (0.437,0.464)  0.02 (-0.02,0.05)  1.21 (1.13,1.30) |
| *Non-resident* | | | |
|  | State 1 (resting) | State 2 (moderately active) | State 3 (traveling) |
| Step mean (km)  Step SD (km)  Angle mean (radians)  Angle concentration | 0.009 (0.009,0.010)  0.007 (0.007,0.008)  -3.11 (-3.24,-2.98)  0.65 (0.56,0.75) | 0.300 (0.25,0.36)  0.384 (0.326,0.453)  -0.02 (-0.52,0.47)  0.17 (0.08,0.27) | 1.123 (1.084,1.164)  0.750 (0.720,0.780)  -0.04 (-0.11,0.03)  1.01 (0.93,1.09) |

Table S4 Temporal budget of HMM behavioural states of the Persian leopards based on GPS relocation data at two phases based on 3-hour data sampling interval for resident and non-resident collared leopards. The behavioural budget is analysed during general and caching phases.

|  | **Caching phase** | | | **General phase** | | |
| --- | --- | --- | --- | --- | --- | --- |
| Behavioural state | State 1 | State 2 | State 3 | State 1 | State 2 | State 3 |
| Resident individuals | 58% | 24% | 18% | 24% | 27% | 49% |
| Non-resident individuals | 58% | 27% | 14% | 26% | 28% | 46% |


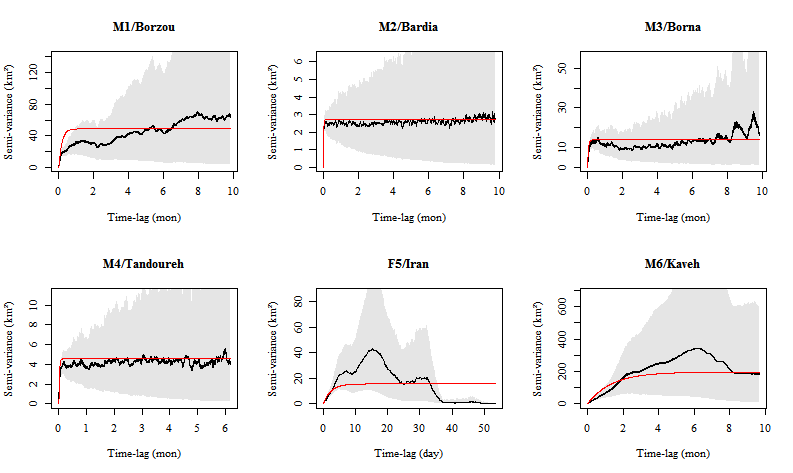


Figure S1 Objective assessment of residency in leopards, based on the variogram of each individual’s observed movement track, obtained from Farhadinia et al. [5], following the workflow described by Calabrese et al. [6] for the R package ‘ctmm’ version 0.4.0. For all individuals, the fraction of the variogram displayed is 80% of the duration of each dataset, except for F5/Iran which shows the entire collaring period, i.e. 54 days.


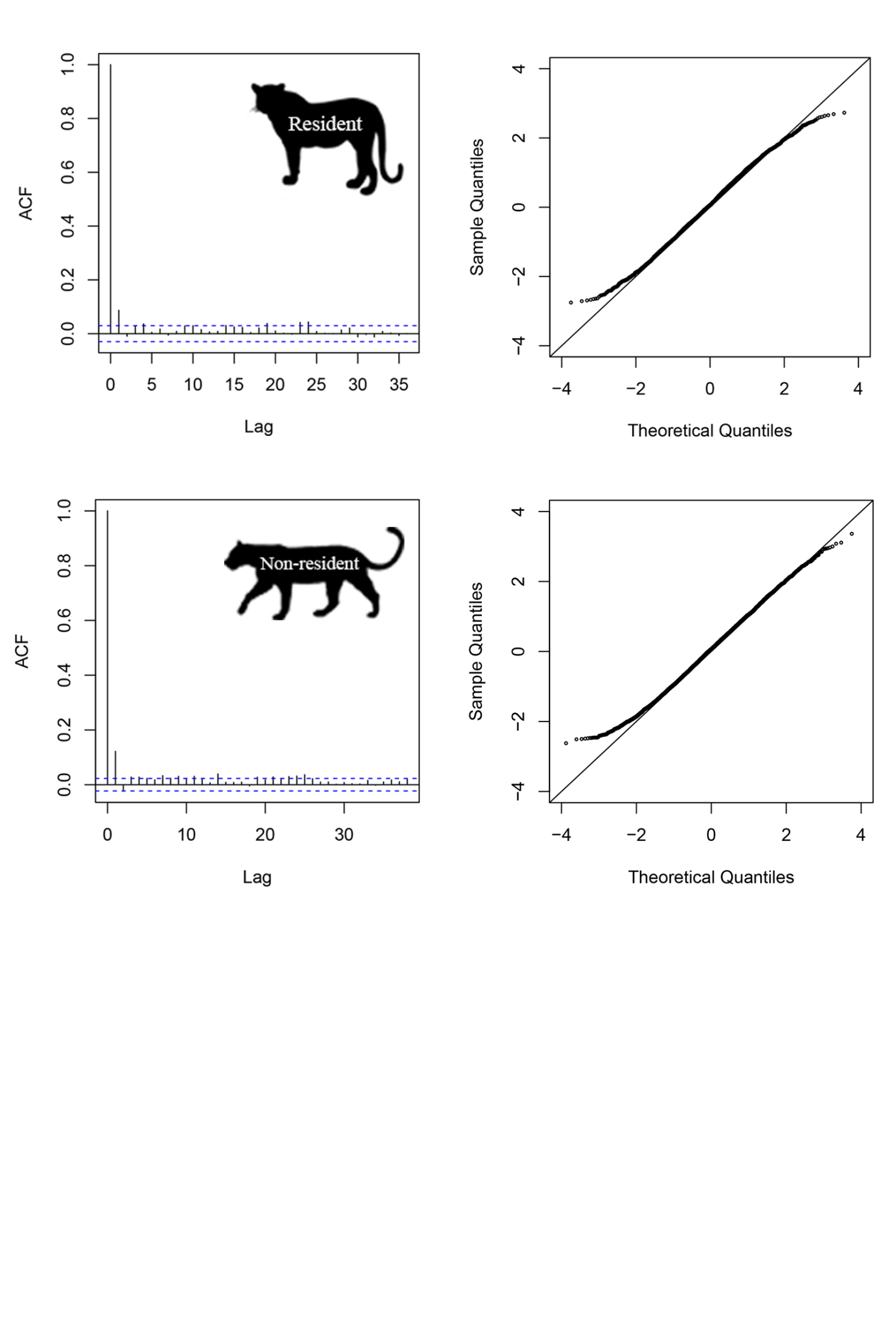


Figure S2 Normal quantile-quantile plot (left), and autocorrelation function plot (right) of the step length pseudo-residuals, for the 1-hour data set during general phase of resident and non-resident individuals.


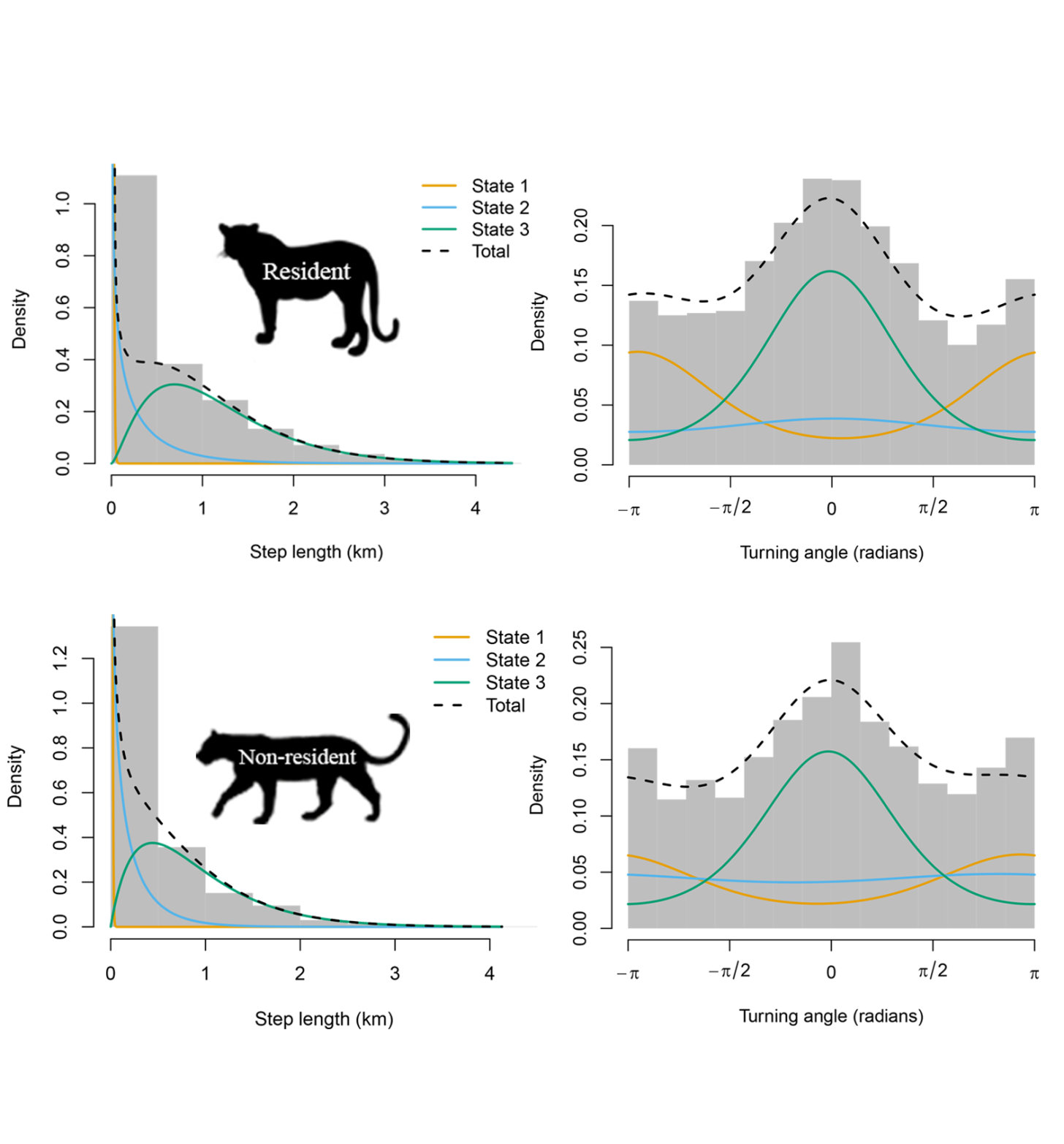


Figure S3 Histograms of observed step lengths (left) and turning angles (right) in the 3-hour data set for general phase of resident and non-resident collared leopards. The colored lines are the estimated densities in each state, and the dotted black line is their sum.


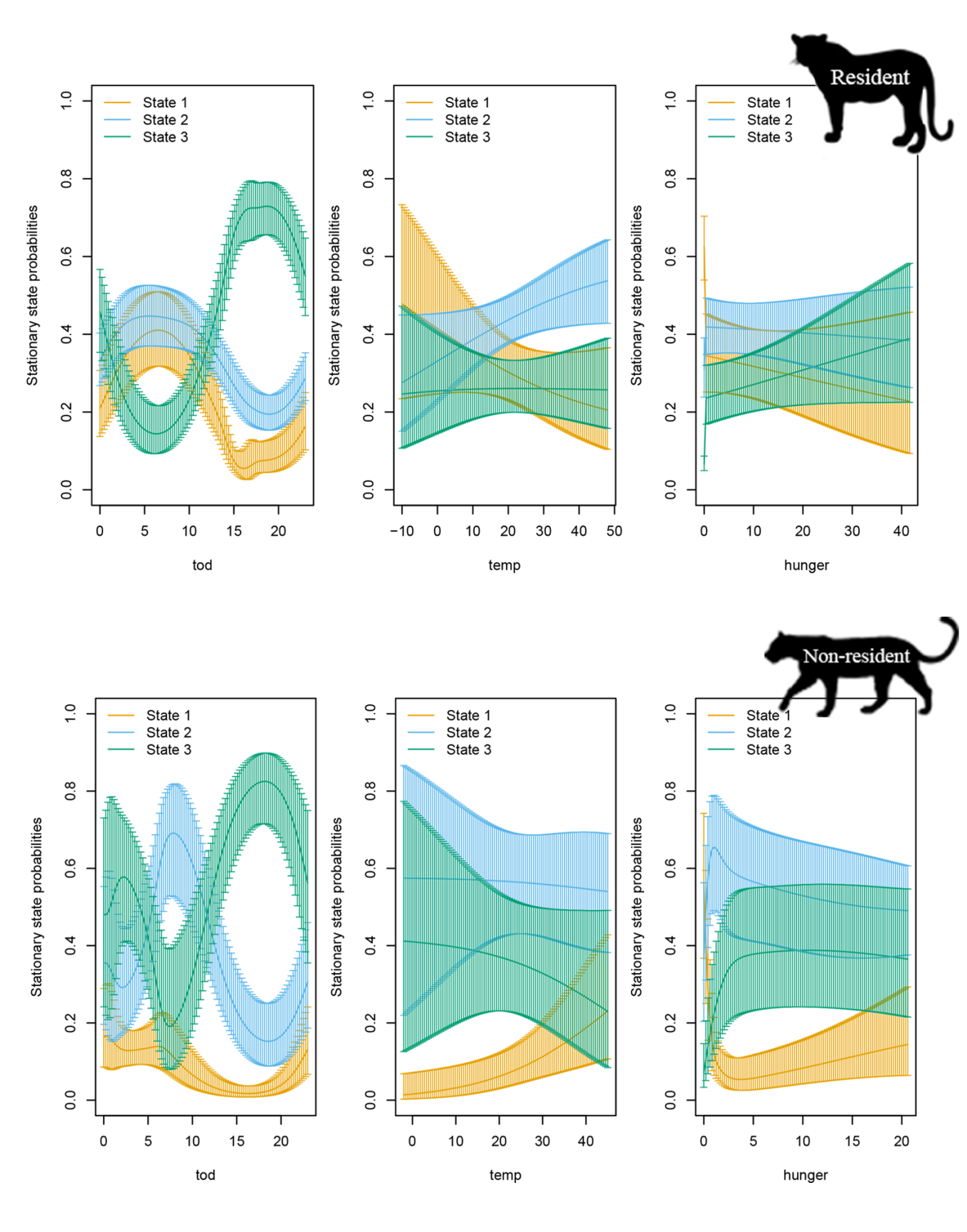


Figure S4 Stationary state probabilities for the 3-hour data set for general phase for resident and non-resident collared leopards, as functions of the three covariates: time of day (left), temperature (middle), and hunger (right). The vertical lines give point wise 95% confidence intervals.


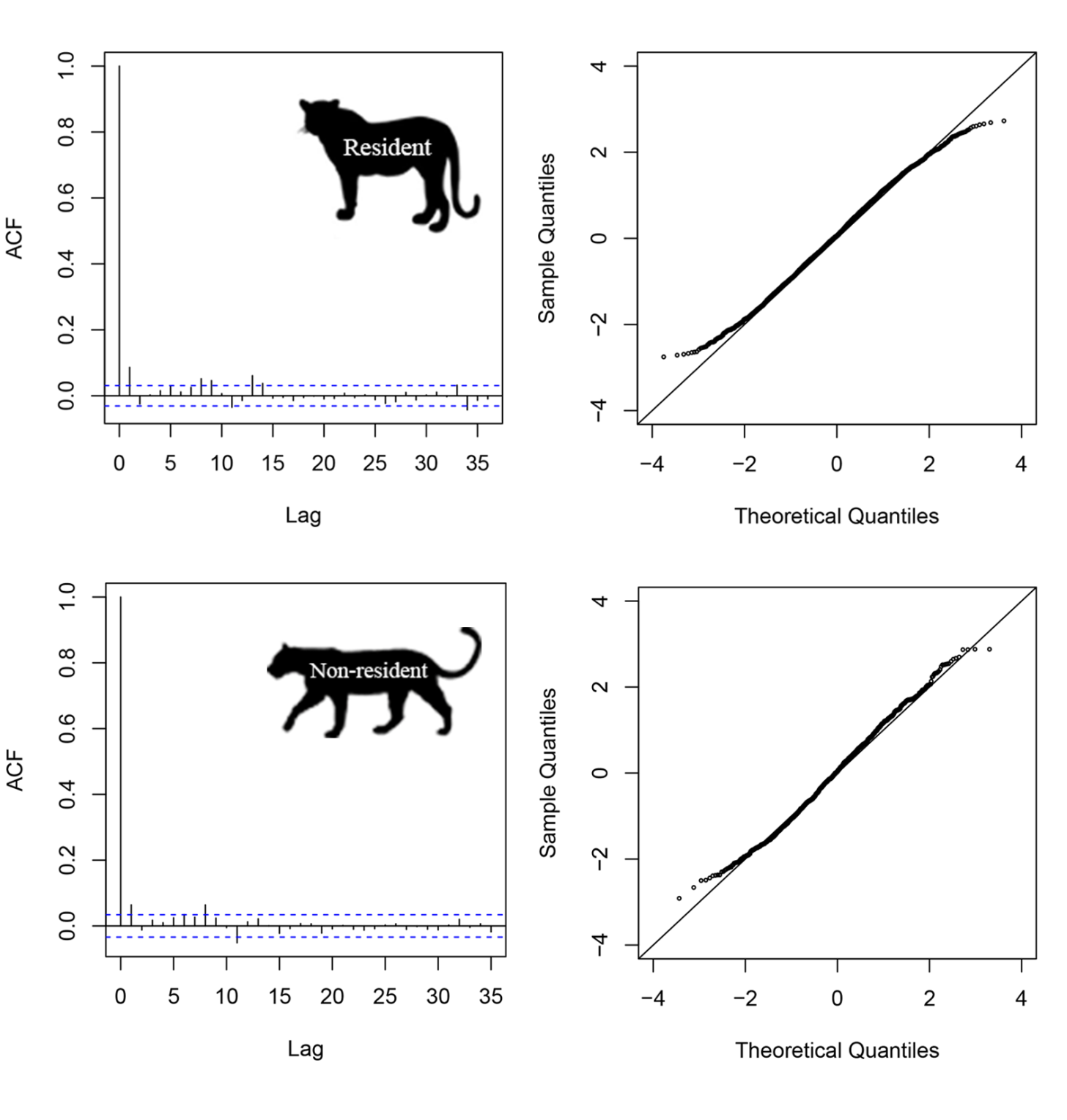


Figure S5 Normal quantile-quantile plot (left), and autocorrelation function plot (right) of the step length pseudo-residuals, for the 3-hour data set during general phase of resident and non-resident individuals.

## References

1. Mukherjee S, Goyal SP, CHELLAM R. Standardisation of scat analysis techniques for leopard (Panthera pardus) in Gir National Park, Western India. Mammalia. 1994;58:139–44.

2. Rezaei A. Niche partitioning between Persian leopard and Asiatic cheetah in Bafq, central Iran. Karaj: University of Tehran; 2014. p. 134.

3. Ackerman BB, Lindzey FG, Hemker TP. Cougar food habits in Southern Utah. JWildlManage. 1984;48:147–55.

4. Ghoddousi A, Soofi M, Kh. Hamidi AK, Lumetsberger T, Egli L, Khorozyan I, et al. Assessing the role of livestock in big cat prey choice using spatiotemporal availability patterns. PLoS One. Public Library of Science; 2016;11:e0153439.

5. Farhadinia MS, Johnson PJ, Macdonald DW, Hunter LTB. Anchoring and adjusting amidst humans: Ranging behavior of Persian leopards along the Iran-Turkmenistan borderland. PLoS One. 2018;13:e0196602.

6. Calabrese JM, Fleming CH, Gurarie E. ctmm: an r package for analyzing animal relocation data as a continuous-time stochastic process. Methods Ecol Evol. 2016;7:1124–32.
